# A prospective, blinded evaluation of a video-assisted ‘4-stage approach’ during undergraduate student practical skills training

**DOI:** 10.1186/1472-6920-14-104

**Published:** 2014-05-22

**Authors:** Katrin Schwerdtfeger, Saskia Wand, Oliver Schmid, Markus Roessler, Michael Quintel, Kay B Leissner, Sebastian G Russo

**Affiliations:** 1 Department of Anaesthesiology, University Hospital Göttingen, Göttingen 37075, Germany; 2 VA Boston Healthcare System, Boston, MA, USA

**Keywords:** Teaching methods, Medical students, Trauma care

## Abstract

**Background:**

The 4-stage approach (4-SA) is used as a didactic method for teaching practical skills in international courses on resuscitation and the structured care of trauma patients. The aim of this study was to evaluate objective and subjective learning success of a video-assisted 4-SA in teaching undergraduate medical students.

**Methods:**

The participants were medical students learning the principles of the acute treatment of trauma patients in their multidiscipline course on emergency and intensive care medicine. The participants were quasi- randomly divided into two groups. The 4-SA was used in both groups. In the control group, all four steps were presented by an instructor. In the study group, the first two steps were presented as a video. At the end of the course a 5-minute objective, structured clinical examination (OSCE) of a simulated trauma patient was conducted. The test results were divided into objective results obtained through a checklist with 9 dichotomous items and the assessment of the global performance rated subjectively by the examiner on a Likert scale from 1 to 6.

**Results:**

313 students were recruited; the results of 256 were suitable for analysis. The OSCE results were excellent in both groups and did not differ significantly (control group: median 9, interquantil range (IQR) 8–9, study group: median 9, IQR 8–9; p = 0.29). The global performance was rated significantly better for the study group (median 1, IQR 1–2 vs. median 2, IQR 1–3; p < 0.01). The relative knowledge increase, stated by the students in their evaluation after the course, was greater in the study group (85% vs. 80%).

**Conclusion:**

It is possible to employ video assistance in the classical 4-SA with comparable objective test results in an OSCE. The global performance was significantly improved with use of video assistance.

## Background

Practical clinical skills are essential requirements for physicians and should be thoroughly learned during medical school training. The demands on the medical schools to teach these skills have increased considerably since the introduction of the new German licensing regulations for physicians [1].

There are a number of concepts regarding the preclinical and clinical care of trauma patients, but teaching these has only played a subordinate role in student training. This changed in 2004 when the 4-stage approach (4-SA) was implemented by the department of anesthesiology in the multidisciplinary emergency and intensive care medicine course as a didactic tool for teaching practical skills in the care of trauma patients to students in small groups [2]. This method has been routinely used for years in international courses on the structured care of trauma patients, e.g. advanced life support (ALS), prehospital trauma life support (PHTLS) and European trauma course (ETC.). Originally, the 4-SA was described by Peyton and has been adapted by Bullock for resuscitation courses in the United Kingdom [3,4]. The 4-SA consists of the following steps:

1. Real-time demonstration of the skill by the instructor.

2. Repetition of the demonstration by the instructor with a detailed explanation.

3. Repetition of the demonstration by the instructor following instructions from a trainee.

4. Repetition of the demonstration by the trainee and practice of the skill by all trainees.

According to Bullock, the 4-SA can facilitate learning of practical skills [3]. The four stages successively transfer the responsibility of performing a skill from the instructor to the trainee. Continuous repetition will lead to a steady improvement of the skill. However, the method is time consuming and personnel-intensive [3]. Several studies have shown that simple technical skills can also be taught with a markedly reduced didactic approach [5,6]. Moreover, the subject matter required for exams should be taught to the entire cohort in a consistent manner in order to achieve successful learning and passing grades in final examinations of modules that are part of the curriculum.

The aim of this study was to evaluate to which extent a video-assisted 4-SA is feasible and whether it can be effectively integrated into a required module of the curriculum. Furthermore, we hypothesized that equally good OSCE test results can be achieved using a video-assisted 4-SA despite reduced personnel requirements.

## Methods

### Participants

All students enrolled in the multidisciplinary curricular course “emergency and intensive care medicine” in the summer semester 2011 and winter semester 2011/2012 were recruited for the study. All students consented to participate in this analysis and to have their test results evaluated for the purposes of this study.

Because there was no patient contact and no patient data was analyzed, the local institutional ethics committee waived the need to formally consider this study as a clinical trail, but did approve to the conductance of the study (number 24/5/13An).

### Intervention

**Figure 1:**
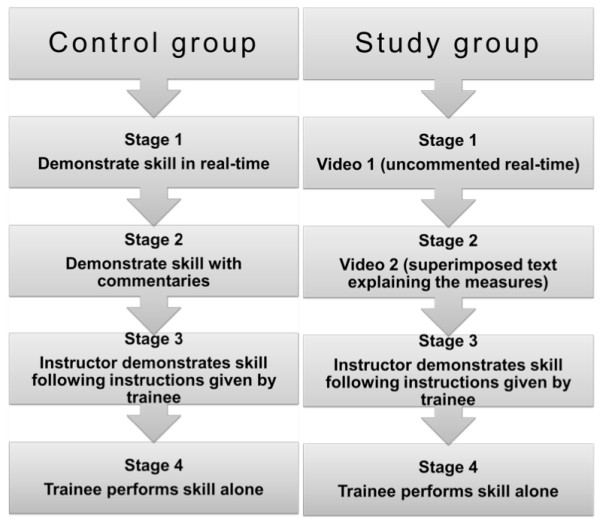
**Flow diagram of control and study groups.** The control groups were taught by the classical 4-Stage Approach. The study groups were taught by a modified 4-stage approach in which the instructor was replaced by a video presentation in steps one and two.

The participating students were assigned by staff of the office of the dean into groups of maximal six students each, totaling 28 groups. 14 of these groups were randomly allotted (randomizer.org) to the study arm and the other 14 to the control arm. The topic of the module was the structured examination of a trauma patient following the ABCDE mnemonic protocol, initially described by N. Caroline [7], as well as integral basic treatment and monitoring (neck immobilization, oxygen administration, insertion of a peripheral venous cannula, monitoring of ECG, pulse oximetry and blood pressure,). The students in the control group were taught using the classical 4-SA during the sixty minutes dedicated for teaching the structured clinical examination. In the study group, the instructor was replaced by video presentations during the initial two steps of the 4-SA. The video of the first step showed the uncommented examination and treatment of the trauma patient in real-time. In the video of the second step, text explaining the procedures was superimposed onto the video images. Steps three and four were conducted in a manner identical to the classic 4-SA (see Figure 1).

### Data collection

At the end of the two-week multidisciplinary course each student was tested in five relevant topics of emergency and intensive care medicine at five distinct OSCE stations by faculty members. In one of these five tests the students had to perform a structured clinical examination of a simulated trauma patient. The faculty examiners (OS and KS) were blinded to the student’s group assignment.

#### Objective test results

The students’ performance was evaluated at the five-minute “trauma care” OSCE station using a checklist of nine dichotomous items (e.g. evaluation of consciousness, palpation of peripheral pulse, auscultation of bilateral lung fields, measurement of capillary refill, abdominal examination), focusing on the steps A, B and C of the ABCDE mnemonic protocol.

#### Subjective opinion of the examiner (assessment of the global performance)

In addition to the checklist, the examiners added a subjective assessment of the student’s global performance using a Likert scale of 1 to 6 (1 = very good, 6 = failed). This served to describe the overall impression of a student’s performance during the clinical examination of a simulated trauma patient, independent of the objective parameters. This global performance evaluation did not influence the student’s final grade.

#### Student’s subjective impression of knowledge gained and free-text comments

The medical school evaluates all courses of the undergraduate curriculum with standardized online surveys regarding the learning outcomes. Pre- and posttest student self-assessments serve to monitor the teaching quality [8]. In addition, students rate their subjective perception of their own acquired skills on a six-point scale (from ‘fully agree’ to ‘not at all true’). In regards to the structured evaluation of pre-hospital trauma patients, students needed to evaluate the following statement: “I am capable to perform a structured evaluation of pre-hospital trauma patients”. In addition, the results of this survey served as an external quality control measure for the present study. Furthermore, post-course, anonymous free-text student comments were analyzed as an additional component for external evaluation. Positive and negative comments about teaching formats and didactical methods for the required course as a whole and separately for the topic of the studied module were tallied. The data from the two semesters prior to the beginning of the study were compared to those of the two semesters of the study.

#### Data analysis and statistical methods

The examination protocols were machine read by the department of information technology of the medical faculty. The data was stored in Microsoft Excel 2010® worksheets and made available to the authors. Statistical analysis was performed with Statistica 10 (StatSoft). All results are presented as median, 25–75 interquartile range (IQR), and were analyzed utilizing the Mann–Whitney U-test.

## Results

A total of 313 students were recruited for the study. Fifty-seven students were excluded from the final analysis (control group 29; study group 27) as the global assessment had not been completed. Of the remaining 256 students, 129 were in the control group (73 female, 56 male) and 127 in the study group (77 female, 50 male).

### Objective checklist evaluation

**Figure 2:**
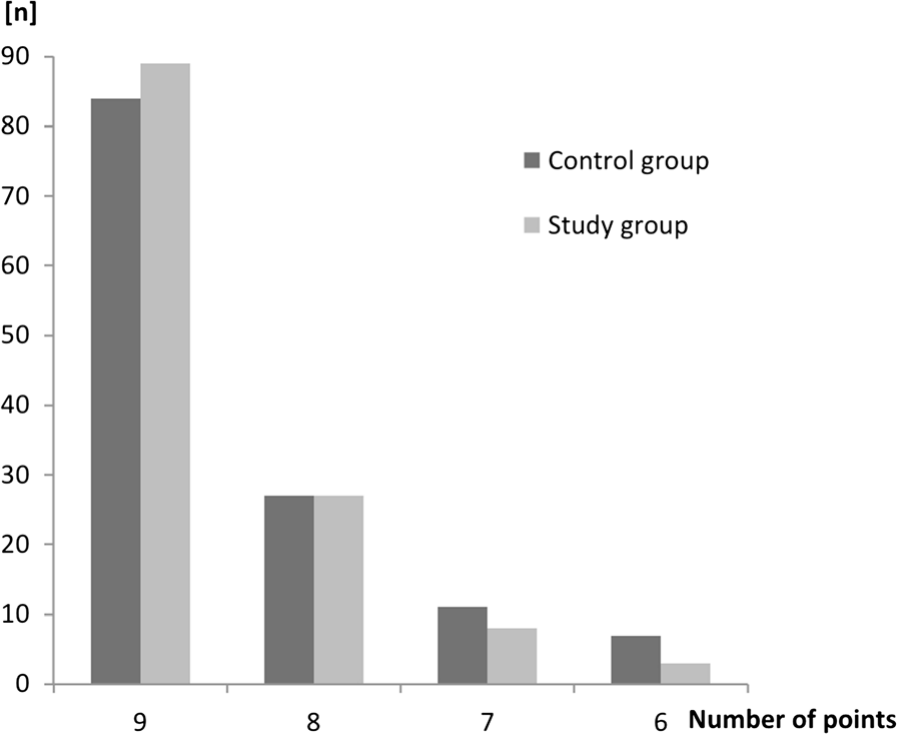
**Number of students for the corresponding number of points achieved during the Objective Structured Clinical Examination (OSCE) presented for the control and study group.** No student achieved less than six of maximum nine points.

The two teaching methods did not differ in the total number of points achieved by the students during the OSCE: Control group median 9, IQR 8–9 points; Study group median 9, IQR 8–9 points (p = 0.29, see also Figure 2).

### Assessment of the global performance

**Figure 3:**
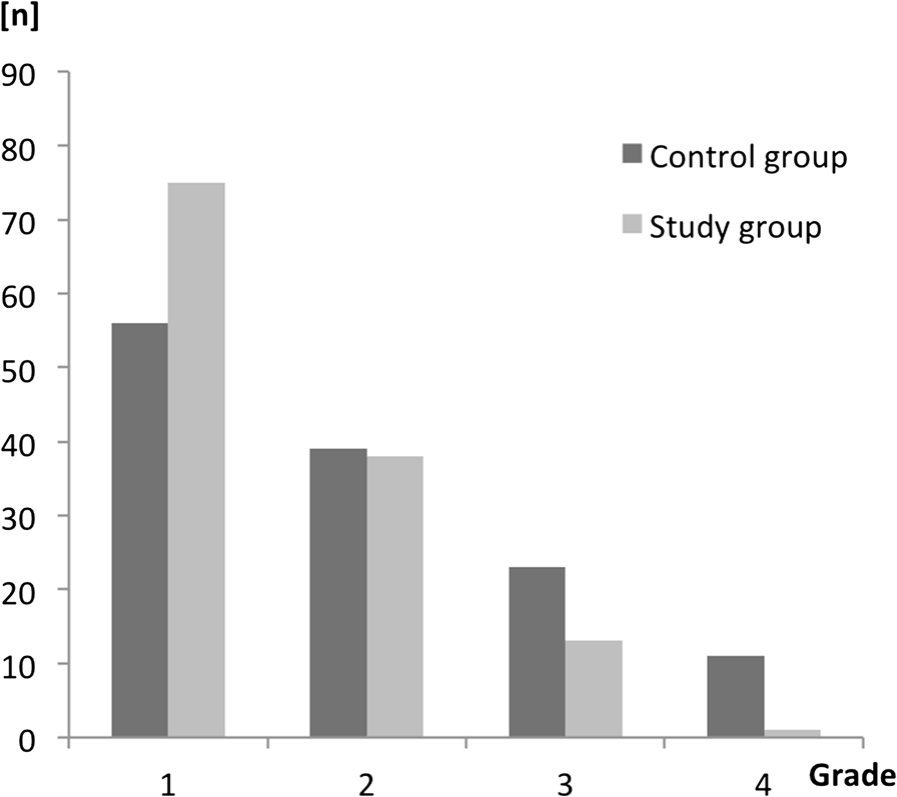
**Grades for the global performance presented for the control and study group as the number of students for the corresponding grade.** No student’s globe performance was rated as less than 4 on a Likard Scale from 1–6.

**Figure 4:**
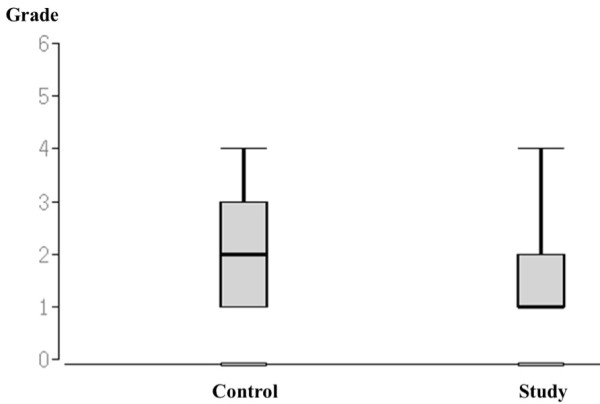
**Grades for the global performance presented for the control and study group.** The Box-plots display the full range, the median and the interquantil range. No student’s globe performance was rated as less than 4 on a Likard Scale from 1–6.

The students in the study group achieved significantly better results regarding the global performance: Control group median 2, IQR 1–3; study group median 1, IQR 1–2; (p < 0.01, see also Figures 3 and 4).

### Knowledge increase and free-text comments

In the semester preceding the first semester of this study, the students subjectively rated their relative increase in knowledge of how to treat a trauma patient at 80%. During the first semester of the study, the relative increase in knowledge was rated at 91% and in the following semester at 80%. The analysis of the free-text comments is shown in Table 1.

**Table 1:** Analysis of the free-text comments at the end of the multidiscipline course

	**Positive**		**Negative**	
	**Before study**	**During study**	**Before study**	**During study**
All comments (n)	61	72	42	40
Course topic “trauma” (n)	1	7	4	1
% regarding “trauma”	1.6%	9.7%	9.5%	2.5%

The free-text comments given as part of the evaluation before and during the semester under study. The comments are divided into positive and negative remarks about the course as a whole as well as those only pertaining to the practical module on trauma management (“trauma”). The number of comments pertaining to trauma management are also given as the percentage of the total number of comments.

### Utilization of resources

Two instructors are required for steps one and two of a 4-SA. The presence of a second instructor is not necessary during the video-assisted presentation of step one and two (approximately 20 minutes in total). Assuming an average of 155 students per semester und a maximum group size of six students (corresponds to 26 groups), this would result in the saving of 520 minutes, which is equal to one man-day.

### Exclusion of data

As the global scoring has not been an integral and compulsory part of the OSCE examination, the assessors occasionally did not complete the global scoring. For good clinical practice we hence excluded all students from further analysis, if the intended data set was incomplete. A separate analysis of the entire data set revealed no difference in the check list analysis (p = 0.69), thus we were able to exclude any bias.

## Discussion

In this study we investigated the feasibility of using a video-assisted 4-SA during student training and evaluated how it might affect the students’ performance in exams.

The objective test performance after the video-assisted 4-SA was similar to that after a conventional 4-SA and hence the video-assisted 4-SA did not improve the objective test performance. Our results are therefore similar to those of Sopka et al. who studied a media-assisted 4-SA for training basic life support (BLS) and showed that media-assisted 4-SA was equivalent to traditional 4-SA in teaching BLS [9].

The global performance assessed by the examiners was significantly improved by the use of the video-assisted 4-SA. Educational psychology might offer an explanation for this improvement in the global performance scores. Although favorable learning conditions had been created in both groups by the multiple repetitions, there was a change of media in the study group; from video in steps one and two to instructor in steps three and four. This has been shown to enhance knowledge acquisition [10].

Videos can be used to show moving images and spoken language and, in addition, a multitude of effects such as superimposed text, animation or slow-motion clips. An optimal observation point can be provided for all viewers so that important details become and remain visible. Even difficult-to-illustrate situations can thus be reproduced with the help of videos. In addition, the videos can be made available to the students before the begin of the course to promote a type of “blended learning” [11]. In our study we used text overlays in the second step of the video-assistet 4-SA. Vester [12] was the first to describe the 4 different learning styles. According to Vester these include the auditive, the visual, the haptic and the intellectual learning type. In this regard, the visual learning type could benefit from changing the acoustic explanation in the control group to the visual overlay in the study group. On the other hand, there is no solid evidence to confirm Vester’s theorie of learning, which has been critiqued in the past [13].

Although there are some pedagogic arguments in favor of a 4-SA, no scientific study has shown an objective advantage of the method. Orde et al. compared the 4-SA to traditional two-step methods (2-SA; “see one, do one”) for teaching the insertion of a laryngeal mask airway. The 2-SA required less time and the performance of the participants was adequate [5]. In another study, Greif et al., who compared the 4-SA and traditional teaching methods for teaching needle cricothyrotomy, came to similar conclusions. Leaving out steps two and three of the 4-SA did not result in a difference of the time required to complete a cricothyrotomy or the number of repetitions required before the participant considered himself adequatelytrained [6]. Furthermore, Jenko et al. compared a 2-SA and a 4-SA for teaching chest compression and showed no difference in the results of the two teaching methods [14]. However, in these three studies the skill that was taught and evaluated may be considered simple and one-dimensional, at least in the teaching model that was applied in the course [5,6,13]. Moreover, teaching the structured clinical care of a trauma patient is a complex process, and to our knowledge there are no studies comparing the 2-SA with the 4-SA in the teaching of complex skills.

The conventional 4-SA is not only time consuming and personnel-intensive but also requires well-qualified instructors to guarantee a consistent level of teaching quality independent of the person teaching. In our study, the teaching video has demonstrated the potential to markedly reduce personnel requirements without negatively affecting student performance compared to the 4-SA with two instructors. Importantly, the video-assisted 4-SA was able to reduce the personnel requirements for each one two-week module by a full one man-day, while simultaneously providing an identical presentation of the subject matter. This calculation is limited to our 4 SA with two instructors. Interestingly, the number of negative free-text comments in the evaluation diminished, and an increase in the number of positive comments on the module on trauma management was noted after our study began. In particular the differing presentation styles and priorities of individual instructors in steps one and two of the 4-SA, which were often criticized before starting the study, were standardized with the help of the videos. Moreover, the differences in the presentation styles in the control group were also reduced since the instructors began using the videos to prepare their own lessons as the videos were available to the faculty instructors before the study began to prepare for the course.

The subjective assessment of the students’ relative knowledge increase differed during the two study semesters. It is important to keep in mind that this subjective assessment depended partly on the extent of the participants’ prior knowledge, which was not determined before the course, and thus the resulting relative increase in knowledge must be interpreted with caution. Our study results offer no explanation for the differing results.

One shortcoming of our study is that there was no objective pre-evaluation determining the previous experience of the individual participants and that there was no longitudinal follow-up. Furthermore, we did not collect data for demographic differences between the study groups except for gender. This information potentially could have been useful in assessing the results of the relative increase in knowledge, and in detecting potential differences in the persistence of the imparted knowledge depending on the teaching method. A further limitation of the study was that due to organizational factors it was not possible to maintain the same interval between intervention (course) and data acquisition (OSCE).

## Conclusions

The comparison of two methods for teaching acute clinical care of trauma patients yielded similar results for the traditional 4-SA and a video-assisted 4-SA with respect to the objective test results. Although less personnel is required the video-assisted 4-SA, this method can be of use in improving the global performance of students in an OSCE. In addition, video-assisted 4-SA may contribute to a more consistent level of teaching quality.

## Abbreviations

ALS: Advanced life support; BLS: Basic life support; ETC.: European trauma course; OSCE: Objective structured clinical examination; PHTLS: Prehospital trauma life support; 4-SA: Four stage approach.

## Competing interests

There are no financial or non-financial competing interests to declare.

## Authors’ contributions

KS, SW and SGR designed the study, analyzed and interpreted the data. KS and SGR drafted the manuscript. OS and KS examined the students during the OSCE. MR helped to interpret the data and to draft the manuscript. KBL helped to draft the manuscript and edited the final version. MQ held drafting the manuscript. All authors read and approved the final manuscript.

## Pre-publication history

The pre-publication history for this paper can be accessed here:

http://www.biomedcentral.com/1472-6920/14/104/prepub
